# Case Report: Fatal copper-associated hepatopathy in a Belgian Shepherd Malinois dog initially investigated as suspected malicious poisoning

**DOI:** 10.3389/fvets.2026.1899864

**Published:** 2026-08-10

**Authors:** Ah-Young Kim, Moonhee Jang, Ji-Su Baek, Bok-Kyung Ku, Kyunghyun Lee

**Affiliations:** 1 Animal Diseases Diagnostic Division, Animal and Plant Quarantine Agency, Gimcheon, Republic of Korea; 2 Toxicology and Narcotics Division, Seoul Institute, National Forensic Service, Seoul, Republic of Korea

**Keywords:** Belgian Shepherd Malinois, case report, copper-associated hepatopathy, dog, ICP-OES, rhodanine staining, sudden death, veterinary forensic pathology

## Abstract

Copper-associated hepatopathy (CAH) is a recognized cause of severe liver injury and acute hepatic decompensation in dogs and may present as chronic hepatitis, cirrhosis, or peracute death. This report describes a fatal case of CAH in a 9-year-old, 30-kg female Belgian Shepherd Malinois dog that was initially investigated as suspected malicious poisoning because of sudden death, tongue protrusion, and bloody feces at the scene. At forensic necropsy, approximately 55 mL of blood-tinged pleural effusion and 10 mL of blood-tinged ascitic fluid were present, the spleen was markedly enlarged, and both kidneys showed multiple dark cortical foci, whereas the liver lacked striking gross abnormalities apart from increased fragility. Histologically, the liver showed multifocal to coalescing hepatocellular degeneration and necrosis with abundant coarse brown intracytoplasmic pigment, raising suspicion of copper accumulation. Rhodanine staining confirmed copper-associated pigment within centrilobular hepatocytes, and toxicologic screening of stomach contents, liver, and kidneys was negative for common exogenous toxicants relevant to malicious poisoning. Quantitative analysis by inductively coupled plasma optical emission spectrometry (ICP-OES) yielded a hepatic copper concentration of 887.4 μg/g wet weight, corresponding to approximately 3176.4 μg/g on a dry-matter basis (estimated, not directly measured) when an assumed hepatic dry-matter content of 28% is applied. This estimated dry-weight concentration exceeds the commonly cited toxic threshold of >1,000 μg/g dry weight and, considered together with the histologic lesions and copper-specific staining, was interpreted as supportive evidence of copper-associated hepatopathy contributing to the fatal outcome. This case highlights CAH as an important differential diagnosis in dogs that die suddenly with hemorrhagic findings, even when the liver is grossly unremarkable and the initial forensic suspicion is malicious poisoning. It also emphasizes the diagnostic value of integrating necropsy, histopathology, copper-specific histochemistry, toxicologic screening, and quantitative copper analysis in veterinary forensic investigations.

## 1 Introduction

Copper-associated hepatopathy (CAH) is a well-recognized metabolic liver disease in dogs characterized by excessive copper accumulation within hepatocytes, resulting in oxidative injury, hepatocellular degeneration, necrosis, chronic hepatitis, cirrhosis, or acute liver failure (1, 2). Breed predispositions have been documented in Bedlington Terriers, Labrador Retrievers, West Highland White Terriers, and Dalmatian dogs, although copper-associated liver disease has been reported in a broader range of breeds (1, 3). Because the clinicopathologic presentation can vary considerably, histopathology and quantitative hepatic copper analysis remain central to diagnosis, classification, and clinicopathologic interpretation (1, 2, 4).

In veterinary forensic practice, sudden and unexpected death accompanied by hemorrhagic signs often raises immediate suspicion of malicious poisoning, leading to police involvement and formal diagnostic investigation (5, 6). In such cases, differentiation between exogenous toxicant exposure and endogenous metabolic or pathologic disease requires a systematic, multidisciplinary approach that incorporates necropsy, histopathology, toxicology, and targeted ancillary testing (7, 8). Previous autopsy-based research has shown that characteristic histopathological changes across multiple organs, interpreted alongside toxicological findings, provide valuable evidence for distinguishing poisoning from alternative causes of death and improving diagnostic accuracy in forensic investigations (9). This distinction is particularly important in companion-animal forensic cases, in which gross lesions alone may be nonspecific and early assumptions of criminal intoxication can influence the direction of the investigation (5–7). Notably, CAH rarely presents as sudden, unexpected death, because most descriptions emphasize chronic hepatitis or cirrhosis rather than peracute fatal decompensation (1, 2, 4); this further underscores the need to differentiate an endogenous metabolic crisis from suspected poisoning using integrated necropsy, histopathology, copper-specific histochemistry, and toxicological analysis (7–9).

The present report describes a fatal case of CAH in a Belgian Shepherd Malinois dog that was initially investigated as suspected malicious poisoning. The case highlights the importance of considering CAH as a differential diagnosis in peracute canine death with hemorrhagic findings and illustrates the value of integrating forensic toxicology, copper-specific histochemistry, and quantitative hepatic copper analysis to establish the cause of death in veterinary forensic casework.

## 2 Case description

A 9-year-old, 30-kg female Belgian Shepherd Malinois dog was found dead at home with the tongue protruding and bloody feces present on the floor. Because of the peracute death and the hemorrhagic appearance of the scene, the owner suspected malicious poisoning and reported the case to the police. The carcass was subsequently submitted to a national veterinary diagnostic laboratory for forensic necropsy to determine the cause of death.

Detailed antemortem clinical information was not available at the time of submission. No history was provided regarding preceding clinical signs, previous medical conditions, medication use, dietary supplementation, or familial liver disease. Vaccination and deworming status were also unknown. In addition, a formal body condition score, the exact postmortem interval, any history of vomiting, the recovery of environmental bait or toxin at the scene, detailed dietary history, and any history of copper supplementation were not available for this case and could not be reconstructed from the submission materials. All identifying information about the owner and animal has been removed from this report.

### 2.1 Necropsy findings

**Figure 1:**
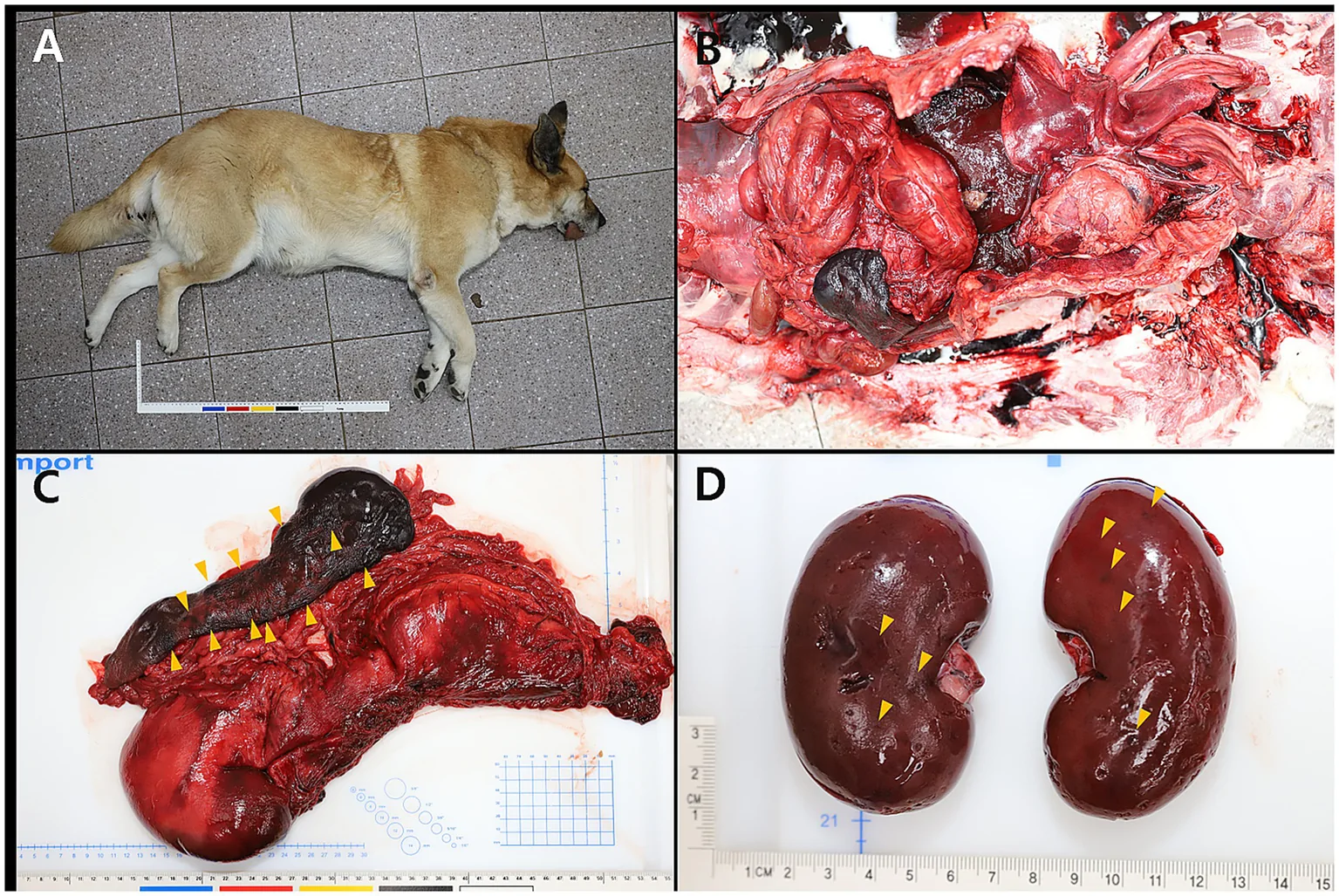
Gross findings at forensic necropsy. **(A)** The Belgian Shepherd Malinois female dog at presentation, with good to moderate body condition. **(B)** Blood-tinged internal organs with hemothorax and hemoabdomen; the liver was fragile on sectioning. **(C)** Severe enlargement of the spleen with multifocal dark red foci (arrowheads). **(D)** Bilateral kidneys showing multifocal dark cortical foci (arrowheads) on the cortical surface.

At necropsy, the carcass was in good to moderate body condition, and postmortem change was within acceptable limits for diagnostic evaluation. Approximately 55 mL of blood-tinged pleural effusion was present in the thoracic cavity, and approximately 10 mL of blood-tinged ascitic fluid was present in the abdominal cavity. The spleen was diffusely enlarged to approximately twice the expected size and had a dark red coloration with mildly rounded margins. Both kidneys contained multiple randomly distributed dark-black foci on the cortical surface (Figure 1).

The liver was grossly unremarkable in overall size and color, although the parenchyma appeared fragile on sectioning; an absolute liver weight was not recorded at necropsy and is therefore acknowledged as a limitation. No gross jaundice or icterus of the mucous membranes, sclerae, or subcutaneous tissues was observed. The gallbladder and major extrahepatic bile ducts were unremarkable. The peripheral and visceral lymph nodes and the pancreas were not specifically described in the necropsy record, and detailed gross findings for these organs are therefore not available. With respect to the gastrointestinal tract, no obvious mucosal erosions or ulcers were recorded in the stomach or intestine despite the hematochezia noted at the time of death; blood was present within the intestinal contents, consistent with that hematochezia. No other striking gross lesions were identified in the thoracic or abdominal organs.

## 3 Diagnostic assessment

### 3.1 Histopathology and special staining

**Figure 2:**
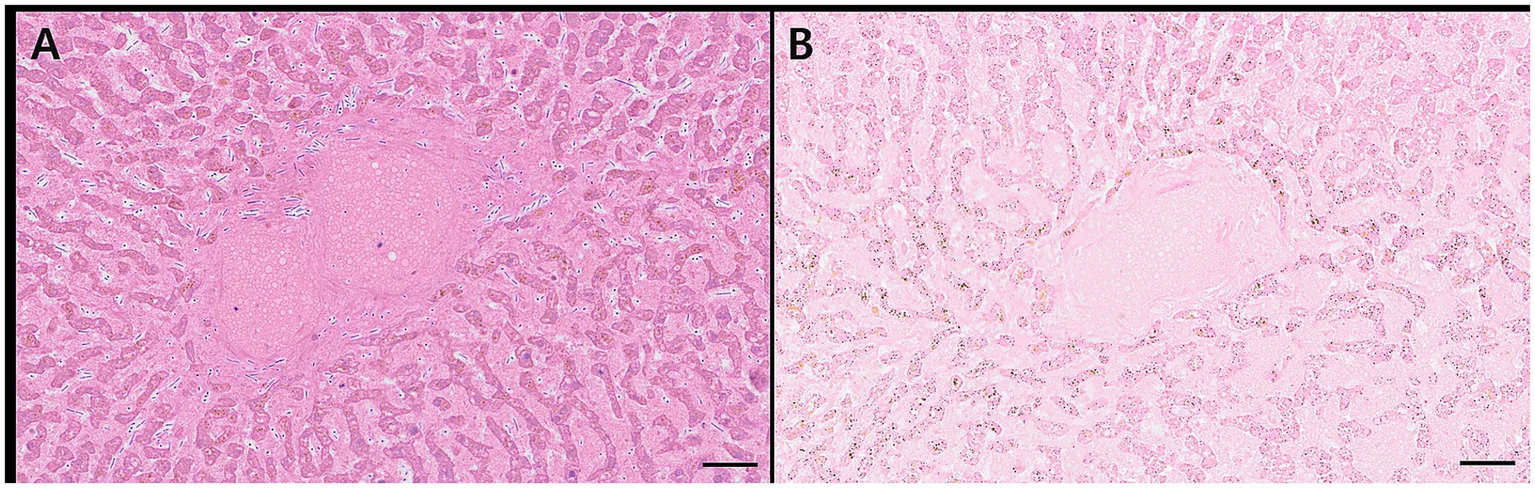
Microscopic findings in the liver: **(A)** Intracytoplasmic accumulation of coarse brown granular pigment associated with hepatocellular degeneration and necrosis in the centrilobular region, with severe congestion of the central vein and sinusoids. Hematoxylin and eosin, original magnification 400×, scale bar = 50 μm. **(B)** Intracytoplasmic copper deposits stained green-black color by rhodanine staining, with a centrilobular distribution. Original magnification 400×, scale bar = 50 μm.

Representative tissue samples were collected at necropsy, fixed in 10% neutral-buffered formalin, routinely processed, embedded in paraffin, sectioned, and stained with hematoxylin and eosin for histopathologic evaluation. Microscopically, the liver exhibited multifocal to coalescing hepatocellular degeneration and necrosis. The necrosis was predominantly centrilobular to mid-zonal in distribution, corresponding to the regions of most prominent pigment accumulation, with relative sparing of periportal hepatocytes. Many hepatocytes contained abundant coarse brown granular intracytoplasmic pigment distributed throughout the hepatic parenchyma and particularly prominent in areas of hepatocellular injury (Figure 2A). The associated inflammatory infiltrate was mild and predominantly mononuclear, comprising lymphocytes and macrophages within portal and lobular regions; a prominent neutrophilic or eosinophilic component was not identified. Fibrosis was limited to mild periportal fibrous expansion (consistent with a low-grade, early-stage change) without established bridging fibrosis or cirrhotic nodular remodeling. Convincing hepatocellular regenerative changes, such as well-defined regenerative nodules or prominent mitotic activity, were not prominent and could not be reliably assessed in the available sections. Additional hepatic changes included scattered bile duct profiles, lobular inflammation, and cholestatic change.

The splenic parenchyma showed cortical tissue necrosis with multifocal to coalescing hemorrhage, resulting in partial to marked effacement of normal splenic architecture by necrotic debris and extravasated erythrocytes. These lesions were interpreted as severe necrohemorrhagic splenic injury associated with marked systemic circulatory or coagulopathic disturbance. In the kidneys, the grossly visible dark cortical foci corresponded microscopically to multifocal cortical hemorrhage with intratubular pigmented casts, interpreted in conjunction with the hepatic and splenic findings as reflecting systemic hypoxia, shock, and pigment nephropathy.

To further characterize the intrahepatocytic brown pigment, copper-specific histochemical staining was performed. Rhodanine staining demonstrated intense green-black granular staining within hepatocytes, with prominent centrilobular distribution, confirming copper-associated intracytoplasmic accumulation (Figure 2B). Perls’ Prussian blue staining for iron was not performed in this case. This is acknowledged as a limitation: although the positive rhodanine reaction supports copper as the predominant intracytoplasmic pigment, the contribution of iron-containing pigment (e.g., hemosiderin) was not specifically excluded by a dedicated iron stain.

### 3.2 Toxicologic screening and quantitative copper analysis

To investigate the owner’s suspicion of malicious poisoning, comprehensive toxicologic screening was performed using stomach contents and tissue samples from the liver. Liquid chromatography–tandem mass spectrometry (LC–MS/MS) and gas chromatography–mass spectrometry (GC–MS) were used to screen for common malicious toxicants, including organophosphates, carbamates, anticoagulant rodenticides, and alkaloids such as strychnine. All tested compounds were below the analytical limits of detection.

Quantitative hepatic copper analysis was performed by inductively coupled plasma optical emission spectrometry (ICP-OES). For sample preparation, 1 mL of liquid sample (or 1 g of solid sample) was placed in a microwave digestion vessel together with 6 mL of nitric acid and 1 mL of hydrogen peroxide. The mixture was digested using a Start D microwave digestion system (Milestone, Sorisole, Italy). The digested sample volume was adjusted to 20 mL with 0.5% nitric acid prior to analysis. ICP-OES analysis was performed on an iCAP 7,000 Series ICP-OES instrument (Thermo Fisher Scientific, Waltham, MA, USA) according to the manufacturer’s recommended operating conditions.

Hepatic copper concentration was 887.4 μg/g on a wet-weight basis. This value was directly measured. Because the published canine reference range and toxic threshold for hepatic copper are conventionally expressed on a dry-weight basis, the result was also expressed on a dry-matter basis. An assumed hepatic dry-matter content of 28% was applied to the wet-weight value (i.e., dry-weight ≈ wet-weight ÷ 0.28), yielding an estimated dry-weight concentration of approximately 3176.4 μg/g dry matter. It should be emphasized that this dry-weight figure is an estimate derived from the directly measured wet-weight value and was not itself directly measured. This estimated dry-weight concentration exceeds the commonly cited canine toxic threshold for hepatic copper accumulation of >1,000 μg/g dry weight and is well above the published canine reference range (<400 μg/g dry weight) (1, 4). The full toxicologic and quantitative results are summarized in Table 1.

**Table 1:** Toxicologic screening and quantitative hepatic copper analysis.

Analyte/parameter	Technique	Result
Organophosphates/carbamates	GC–MS/LC–MS/MS	Below limit of detection
Anticoagulant rodenticides	LC–MS/MS	Below limit of detection
Alkaloids (strychnine, etc.)	LC–MS/MS	Below limit of detection
Hepatic copper concentration	ICP-OES	887.4 μg/g wet weight (≈3176.4 μg/g dry matter, estimated)*; exceeds the commonly cited toxic threshold (>1,000 μg/g dry weight)

The wet-weight concentration was directly measured by ICP-OES. The dry-matter equivalent is an estimate derived from the wet-weight value assuming a hepatic dry-matter content of 28% (i.e., dry-weight ≈ wet-weight ÷ 0.28) and was not directly measured; it should be replaced with a laboratory-measured value if available. Published canine hepatic copper reference range: <400 μg/g dry weight; commonly cited toxic threshold for toxic accumulation: >1,000 μg/g dry weight (1, 4). The estimated dry-weight value of approximately 3176.4 μg/g exceeds this threshold.

## 4 Discussion

This case illustrates a recurring diagnostic challenge in veterinary forensics, namely distinguishing malicious exogenous poisoning from an endogenous metabolic crisis in dogs that die suddenly with hemorrhagic signs (5). The peracute death of this Belgian Shepherd Malinois, together with hematochezia and a protruding tongue, closely mimicked acute toxicosis and therefore justified immediate police involvement and a full forensic investigation (6, 7). Systematic exclusion of common rodenticides, insecticides, and criminal alkaloids by LC–MS/MS and GC–MS shifted the diagnostic focus away from exogenous poisoning and toward an underlying metabolic hepatopathy (8).

The diagnosis of CAH in this dog was established by integrating histopathology, copper-specific histochemistry, and quantitative hepatic copper analysis, in line with current diagnostic recommendations (1, 2, 4). Routine hematoxylin and eosin staining revealed multifocal to coalescing hepatocellular degeneration and necrosis with abundant coarse intracytoplasmic brown pigment distributed throughout the hepatic parenchyma. Visual identification of intracytoplasmic brown pigment alone is insufficient because copper may be confused with hemosiderin or lipofuscin; copper-specific histochemical staining is therefore considered essential (1). In this case, rhodanine staining demonstrated intense bright red-orange granules predominantly in centrilobular hepatocytes, confirming that the pigment represented copper-bound complexes rather than other pigments.

The quantitative result requires careful interpretation. A hepatic copper concentration of 887.4 μg/g wet weight, corresponding to an estimated 3176.4 μg/g dry matter when an assumed 28% hepatic dry-matter content is applied, drastically exceeds the published canine reference range (<400 μg/g dry weight) and the commonly cited diagnostic and toxic thresholds for hepatic copper accumulation, which are variously reported in the range of approximately >1,000 to 2,000 μg/g dry weight (1, 2, 4, 10). It is important to emphasize, however, that only the wet-weight concentration was directly measured; the dry-weight value is an estimate that is dependent on the assumed hepatic dry-matter fraction and would change if the true dry-matter content differed from the assumed 28%. Hepatic copper concentrations in this range have been reported in dogs with histologically confirmed copper-associated chronic hepatitis (1, 2, 4). Taken together with the marked histologic injury and the centrilobular rhodanine-positive copper accumulation, the quantitative result is interpreted as supportive evidence of copper-associated hepatopathy contributing to the fatal outcome, rather than as definitive proof of frank copper toxicosis, particularly given the estimated nature of the dry-weight value.

The extrahepatic lesions observed in this dog may be interpreted in the context of terminal acute hepatic dysfunction (4, 11). Marked splenomegaly, blood-tinged pleural and peritoneal effusions, and necrohemorrhagic splenic lesions may reflect systemic circulatory disturbances and coagulopathy associated with acute hepatic dysfunction. Massive hepatocellular injury and impaired hepatic synthetic function can predispose to disseminated intravascular coagulation, reduced synthesis of coagulation factors, and subsequent hemorrhage in highly vascular organs such as the spleen; however, this interpretation remains inferential because coagulation parameters (e.g., prothrombin time, activated partial thromboplastin time, platelet count, and fibrinogen) were not available for this case. Similarly, the renal lesions—dark cortical foci that microscopically corresponded to multifocal cortical hemorrhage with intratubular pigmented casts—may reflect pigment nephropathy related to intravascular hemolysis and systemic hypoxia (12), rather than representing a definitively established mechanism. Such a systemic cascade may progress to terminal shock and secondary hypoxic injury, potentially culminating in sudden death (11), although the absence of coagulation testing limits the certainty of this proposed pathophysiologic sequence.

An important feature of this case is the discrepancy between gross and microscopic hepatic findings. At necropsy, the liver appeared grossly unremarkable apart from increased fragility, and no obvious nodular remodeling or striking discoloration was detected, yet histopathology revealed severe, multifocal to coalescing hepatocellular degeneration and necrosis. This discrepancy is an instructive teaching point: severe microscopic hepatic injury may be present despite relatively subtle or nonspecific gross changes, and CAH and severe hepatocellular necrosis may therefore be overlooked when necropsy relies solely on macroscopic assessment (2, 4). Routine histopathology and, in particular, recognition of intracytoplasmic pigment compatible with copper are crucial in similar forensic cases. These observations are consistent with previous reports indicating that dogs with copper-related hepatopathy may lack characteristic gross changes until advanced stages or acute decompensation (2, 4).

Breed-related susceptibility is another point of interest (1–3). Bedlington Terriers, Labrador Retrievers, West Highland White Terriers, and Dalmatian dogs are among the best-characterized breeds with increased risk of CAH, and additional breeds have been reported to exhibit abnormally high hepatic copper concentrations (1–3). To the best of our knowledge, this is the first published description of fatal CAH in a Belgian Shepherd Malinois confirmed by integrated histopathology, copper-specific histochemistry, and quantitative copper analysis. Documenting CAH in this breed expands the spectrum of affected dogs and emphasizes that copper-related liver disease can occur in a broader range of large working breeds than traditionally recognized (2, 3).

The peracute, fatal presentation in this 9-year-old dog also merits comment. Canine copper-associated hepatopathy is frequently a chronic and clinically silent process, in which hepatic copper accumulates gradually and overt clinical signs may be absent or only intermittent until hepatic functional reserve is exhausted or acute decompensation supervenes (1, 2, 4). Within this framework, the lethal manifestation of disease for the first time at an advanced age can be interpreted as terminal decompensation of previously unrecognized, slowly progressive copper-associated liver injury, rather than as the abrupt onset of a new process. It is important to emphasize, however, that the apparent absence of prior clinical signs in this case cannot be confirmed, because no antemortem medical history, prior physical examinations, or serum biochemistry were available; subclinical or intermittent gastrointestinal or hepatic signs may therefore have gone unrecognized or undocumented. For the same reason, a congenital or inherited primary copper-storage disorder was neither established nor excluded in this animal.

This case has several additional limitations. No genetic or molecular testing for copper metabolism-associated variants was performed; accordingly, the underlying mechanism of copper accumulation could not be characterized at the molecular level, and the case cannot distinguish an inherited (primary) copper-storage disorder from secondary or acquired copper accumulation, including a possible dietary or environmental contribution. In addition, as noted above, antemortem clinical history and clinicopathologic data (including serum biochemistry and coagulation parameters), an absolute liver weight, a Perls’ Prussian blue iron stain, and a directly measured hepatic dry-matter content were not available, and the dry-weight copper concentration is therefore an estimate. These limitations should be considered when interpreting the cause-of-death determination, and future cases would benefit from genetic testing and, where feasible, more complete antemortem and postmortem data.

## 5 Conclusion

This case provides, to the best of our knowledge, the first documented description of a fatal metabolic crisis associated with copper-associated hepatopathy (CAH) in a Belgian Shepherd Malinois dog. The clinical and forensic presentation of sudden, peracute death accompanied by prominent hemorrhagic findings closely mirrored the classical scenario of malicious exogenous poisoning. This initially led to criminal suspicion and law-enforcement involvement. However, a systematic, multidisciplinary diagnostic approach—integrating macroscopic necropsy, histopathology, copper-specific rhodanine staining, comprehensive toxicologic screening, and quantitative ICP-OES analysis—successfully excluded external malicious agents and confirmed an endogenous metabolic cause of death.

Crucially, this case demonstrates that severe, fatal CAH can occur even in the absence of striking macroscopic hepatic lesions such as cirrhosis or distinct color changes, highlighting that relying solely on gross necropsy may lead to misdiagnosis. Furthermore, identifying CAH in a Belgian Shepherd Malinois expands the known breed spectrum susceptible to this condition, suggesting that veterinary pathologists and clinicians should maintain a high index of suspicion for copper-related metabolic diseases in a wider variety of large working breeds than traditionally recognized. Ultimately, this report underscores the vital role of ancillary diagnostics and objective toxicologic analysis in veterinary forensic pathology, ensuring accurate cause-of-death determination and preventing erroneous assumptions of criminal intoxication. Taken together, the integrated necropsy, histopathologic, histochemical, and toxicologic findings strongly support CAH as the cause of, or a major contributor to, death, rather than constituting absolute proof. This balanced interpretation is appropriate because the dry-weight hepatic copper concentration was estimated from the measured wet-weight value rather than directly measured, and because coagulation data that would have helped characterize the terminal hemorrhagic process were unavailable.

## Data Availability

The data used and/or analysed during the current study are available from the corresponding author upon reasonable request.
